# An integrated modelling framework for neural circuits with multiple neuromodulators

**DOI:** 10.1098/rsif.2016.0902

**Published:** 2017-01

**Authors:** Alok Joshi, Vahab Youssofzadeh, Vinith Vemana, T. M. McGinnity, Girijesh Prasad, KongFatt Wong-Lin

**Affiliations:** 1 School of Computer Science, University of Manchester, Manchester, UK; 2 Division of Neurology, Cincinnati Children's Hospital Medical Center, Cincinnati, OH, USA; 3 Intelligent Systems Research Centre (ISRC), University of Ulster, Derry–Londonderry, UK; 4 Computer Science and Engineering, Indian Institute of Technology (IIT) Jodhpur, Jodhpur, India; 5 College of Science and Technology, Nottingham Trent University, Nottingham, UK

**Keywords:** computational neural circuit models, neuromodulators, neuropharmacology, orexin/hypocretin, serotonin, norepinephrine/noradrenaline

## Abstract

Neuromodulators are endogenous neurochemicals that regulate biophysical and biochemical processes, which control brain function and behaviour, and are often the targets of neuropharmacological drugs. Neuromodulator effects are generally complex partly owing to the involvement of broad innervation, co-release of neuromodulators, complex intra- and extrasynaptic mechanism, existence of multiple receptor subtypes and high interconnectivity within the brain. In this work, we propose an efficient yet sufficiently realistic computational neural modelling framework to study some of these complex behaviours. Specifically, we propose a novel dynamical neural circuit model that integrates the effective neuromodulator-induced currents based on various experimental data (e.g. electrophysiology, neuropharmacology and voltammetry). The model can incorporate multiple interacting brain regions, including neuromodulator sources, simulate efficiently and easily extendable to large-scale brain models, e.g. for neuroimaging purposes. As an example, we model a network of mutually interacting neural populations in the lateral hypothalamus, dorsal raphe nucleus and locus coeruleus, which are major sources of neuromodulator orexin/hypocretin, serotonin and norepinephrine/noradrenaline, respectively, and which play significant roles in regulating many physiological functions. We demonstrate that such a model can provide predictions of systemic drug effects of the popular antidepressants (e.g. reuptake inhibitors), neuromodulator antagonists or their combinations. Finally, we developed user-friendly graphical user interface software for model simulation and visualization for both fundamental sciences and pharmacological studies.

## 1. Introduction

Neuronal activities, through the firing of action potentials and synaptic transmissions, can be modulated by endogenous neurochemicals called neuromodulators, acting through biophysical and biochemical processes [1,2]. These neuromodulators are released by a distinct population of neurons, and the neuromodulators act on specific receptors which are distributed throughout the brain [3]. Major neuromodulators include serotonin, dopamine, norepinephrine (NE; or noradrenaline), acetylcholine, orexin (or hypocretin), endorphins and octopamine [3]. As a consequence of neuromodulation, neural circuit function can be altered, which in turn can affect cognition, mood and behaviour [3]. In neuropharmacological drug treatment of neurological and neuropsychiatric illnesses, the monoaminergic systems (especially that of serotonin, dopamine and NE) are often targeted [4]. These are achieved, for example, by altering the affinity of the associated receptors that influences the release and reuptake mechanism of the monoaminergic systems [5,6]. As neuromodulators can also influence the biophysical properties of the neurons and synapses via multiple receptors with differential affinities, the complexity level in a neuronal circuit function can be substantial [7,8]. Experimental work often focuses on a specific brain region or system (e.g. certain receptor subtype) or employs a specific experimental methodology specific to the single level of biological organization (e.g. whole-cell recording at the neuronal level or voltammetric recording at specific brain region). Thus, it is difficult to reconcile their systemic implications.

Sufficiently realistic computational neural models can help us to integrate various data types from different studies, and can also generate testable predictions. However, modelling the detailed biophysical effects of neuromodulators can be complex and computationally costly [9–11]. In particular, neuromodulation often involves intracellular signalling processes at the pre-synaptic and post-synaptic sites, and can subsequently affect neuronal firing activities [12,13]. Hence, such computational models that incorporate these biological processes can be time-consuming to develop, and with the multiple model parameters and equations, computationally intensive to evaluate while posing a considerable challenge in scalability.

In this work, we propose a novel neural circuit modelling framework to circumvent such difficulties. To develop a scalable model, we make use of neural population-averaged activity (mean-field like) or firing-rate-type models that describe how the neural population activities depend on the averaged effective neuromodulator-induced currents. The latter are determined by neuromodulators' concentration levels and the corresponding receptor affinities. Compared with other more abstract population-averaged firing-rate-type models [14–17], our model parameters describing the input–output functions and temporal dynamics are informed and constrained by data integrated from a variety of experiments, which include electrophysiology, neuropharmacology, radioimmunoassay, voltammetry and microdialysis.

We discuss such modelling approach in the context of developing and simulating a neural circuit model interconnected among the dorsal raphe nucleus (DRN), locus coeruleus (LC) and lateral hypothalamus areas (LHA), which are major sources of the important neuromodulators serotonin (5-HT), NE and orexin, respectively. The motivations for selecting these brain systems to model are that they are important in regulating physiological functions especially in arousal, are known to interact mutually with each other, and are the targets of several drugs [13,18–21]. In particular, the neuropeptide Ox is known to play an important role in energy homeostasis, food intake and appetite regulation, neuroendocrine functions and sleep–wake regulation [22–24]. The monoamine NE is suggested to be responsible for numerous functions, including stress response, attention, emotion, motivation, decision-making, learning, memory and regulation of sleep (e.g. REM) [25–30], whereas the monoamine 5-HT can affect several physiological functions that include eating behaviour, emotion, and sleep regulation [31–33]. Abnormal 5-HT or NE levels are implicated in mood disorders and anxiety [18,34].

**Figure 1.**
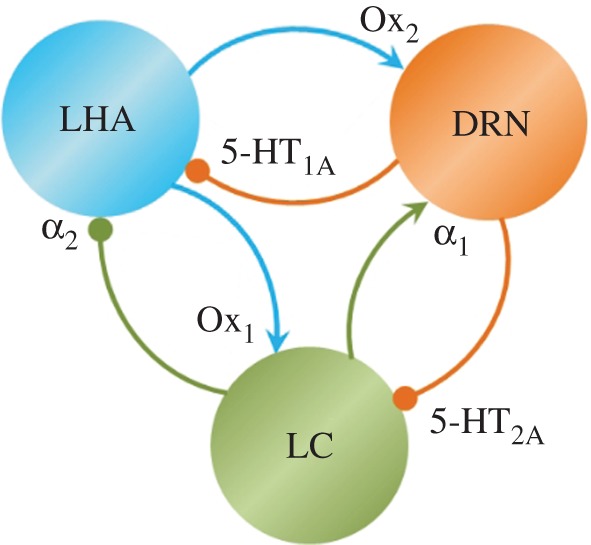
LHA, DRN and LC interactions. Arrows: effective excitatory connections between any two areas; circles: inhibitory connections. Different colours denote different brain areas and their respective connection types and the targeted areas [27–31,35].

The overlapping roles of these neuromodulators are not surprising, given their mutual interconnectivity, and any targeted neurons could themselves be sources of neuromodulators (figure 1). We have also purposefully selected the Ox system as a case study to demonstrate how we can model a neural system that may not be well characterized (when compared with NE).

A comprehensive, biologically faithful, yet efficient computational model at the neural circuit level would enable us to conveniently evaluate, account and predict, at a systems level, important measurable variables such as the concentration levels of the neuromodulators, the neural population firing rate activities, the effects of individual or combined drugs (e.g. reuptake inhibitors or antagonists) and their interdependencies.

The organization of the rest of the paper is as follows. We first describe the general modelling framework. Then, as an example, we demonstrate the steps in modelling three mutually interacting brain regions and discuss the simulation results including drug effects. Next, we describe our user-friendly software for simulating and visualizing the behaviour of such models. Finally, we summarize the results and discuss the implications of this study.

## 2. Results

### 2.1. An integrated modelling framework

**Figure 2.**
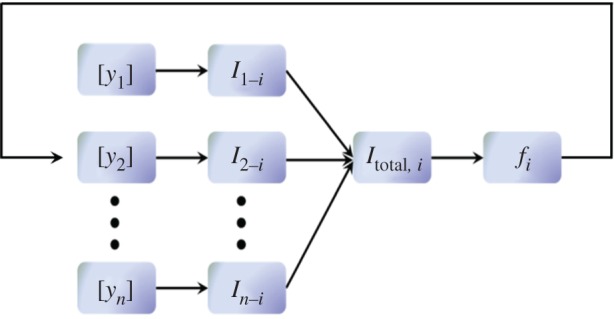
Incorporating afferent currents from neuromodulator concentration levels. [*y*_1_] … [*y_n_*] denote the different neuromodulator concentrations. *i* represents a particular targeted brain region. 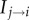 is the corresponding induced currents to region *i*. 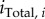 is the total afferent current and *f_i_* is the firing frequency in region *i*. For example, [*y*_1_] and [*y*_2_] may be the concentration levels of serotonin [5-HT] and norepinephrine [NE], and *i* can be the lateral hypothalamus LHA. The big arrow denotes ‘closing the loop’ in the modelling process.

To develop a biologically compatible neural circuit model would require knowledge of electrophysiological properties of the composing neurons in a specific brain region, and the nature of the interactions among themselves and with other neuronal groups. This can include neurons which themselves release neuromodulators. Modelling the release-and-reuptake/decay dynamics of the extracellular concentrations of the neuromodulators would require information inferred from *in vivo* voltammetry or microdialysis studies at the targeted sites under neuronal stimulation. We would also need to know how the variation in neuromodulator concentration can in turn affect neural firing rate activities via neuromodulator-induced currents, hence requiring knowledge of firing rate–neuromodulator concentration or firing rate–current relationships (figure 2). These neuromodulator-induced currents typically involve relatively slow metabotropic G-protein-coupled receptor (GPCR) types, e.g. G-protein-coupled inwardly rectifying potassium (GIRK) or transient receptor potential (TRP) type cation currents [36,37], on targeted neurons, which alter the neuronal firing rate activities. As discussed, explicitly modelling such signalling pathway mechanisms can be complex and computationally intensive if large-scale neural circuits are involved. To circumvent such challenges, we turn to phenomenological yet biologically faithful models to mimic the overall effects.

To begin the modelling process, we first model the neural activity for each brain region using neural population-averaged activity [38]. Because the time constant of the typical neural population firing-rate dynamics is approximately 10–100 ms, it is much faster than the dynamics owing to neuromodulators, which is approximately seconds to minutes (table 1). Hence, we shall ignore the neural population dynamics and assume the system to be dominated by the slower neuromodulator-induced dynamics [57]. In general, different neuronal types can respond differently (in terms of firing rate activity) to the same current injection. In experiments, such relationship is demonstrated by the frequency–current (*f* − *I*) relationship. In a similar vein, we can describe the *i*th neural population by the population firing rate–current (*f* − *I*) curve or input–output function [58]:2.1
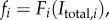
where *f_i_* is the population firing rate, *F_i_* is the input–output function and *I*_total*,i*_ is the total averaged afferent current. Under typical physiological ranges, it suffices to use a threshold-linear function [58]:2.2
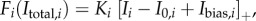
where [*x*]_+_ = *x* if *x* > 0, and 0 otherwise. *K_i_* is the constant gain or slope of the input–output function, *I*_0*,i*_ is the threshold current for non-zero firing and *I*_bias,*i*_ is the current coming from other brain areas. Thus, after a specific threshold value of the averaged afferent current, the neural population will be activated, and there is a linear relationship between the neural firing rate and the overall afferent current. We shall later show that this function fits the experimental data for Ox, 5-HT and NE neurons.

**Table 1.** Basal firing rate, neurotransmitter levels, dynamical time constants, and other model parameters for the LHA–DRN–LC circuits. Asterisk: [39], assuming *V*_max_, and per stimulus release at dorsal lateral geniculate (DLG) and LC will be same. Hash: [40]. Plus: parameter values are tuned to obtain the basal values close to those in experiments.

parameter	description	value	reference, remarks
*f*_DRN_	basal firing rate of 5-HT neurons in DRN	0.8 Hz	[41], *in vitro*
*f*_LC_	basal firing rate of NE neurons in LC	2.15 Hz	[42], *in vitro*
*f*_LHA_	basal firing rate of Ox neurons in LHA	2.3 Hz	[37], *in vitro*
*k*_LHA_	gain of the input–output function for LHA neurons	0.2 Hz pA^−1^	[43]
*K*_DRN_	gain of the input–output function for DRN neurons	0.033 Hz pA^−1^	[10,44,45]
*k*_LC_	gain of the input–output function for LC neurons	0.058 Hz pA^−1^	[46]
*I*_0*,*LHA_	threshold current for non-zero firing of LHA neurons	0 pA	[43]
*I*_0*,*DRN_	threshold current for non-zero firing of DRN neurons	24.82 pA	[10,44,45]
*I*_0*,*LC_	threshold current for non-zero firing of LC neurons	0.028 pA	[46]
*I*_bias,LHA_	afferent current to LHA neurons	11.5 pA	[43]
*I*_bias,DRN_	afferent current to DRN neurons	24.82 pA	[10,44,45]
*I*_bias,LC_	afferent current to LC neurons	37.41 pA	[46]
[*5-*HT]_LHA_	basal [5-HT] level in Ox neurons	1.6 nM	[47]
[5*-*HT]_LC_	basal [5-HT] level in Ne neurons	6.7 fM min^−1^ approximately 0.11 fM s^−1^	[48]
[NE]_DRN_	basal [NE] level in 5-HT neurons	500 pg mg^−1^ approximately 2.95 µM	[49], assuming baseline 5-HT levels at dorsal and rostral raphe are same
[NE]_LHA_	basal [NE] level in Ox neurons	0.83 nM	[50], assuming baseline NE levels at hypothalamus and LHA are the same
[Ox]_DRN_	basal [Ox] level in 5-HT neurons	10 pg mg^−1^ approximately 3.4 nM	[51], assuming baseline Ox level at pons and DRN are the same
[Ox]_LC_	basal [Ox] level in Ne neurons	2 pg mg^−1^ 0.56 nM	[51]
*τ*_[5-HT]_ → _LHA_	time constant of the effect of [5-HT] on Ox neurons	2 s	[36]
*τ*_[5-HT]_ → _LC_	time constant of the effect of [5-HT] on Ne neurons	20 s	[52]
*τ*_[Ox]_ → _DRN_	time constant of the effect of [Ox] on 5-HT neurons	60 s	[53]
*τ*_[Ox]_ → _LC_	time constant of the effect of [Ox] on Ne neurons	20 s	[54]
*τ*_[NE]_ → _DRN_	time constant of the effect of [NE] on 5-HT neurons	20 s	[55]
*τ*_[NE]_ → _LHA_	time constant of the effect of [NE] on Ox neurons	1 s	[56]
*V*_max,[NE]_ → _LC_	maximum uptake rate for the [NE] release in LC neurons	74 nM s^−1^	*
*K*_m,[NE]_ → _LC_	substrate concentration for the [NE] release in LC neurons	400 nM	*
*V*_max,[NE]_ → _DRN_	maximum uptake rate for the [NE] release in DRN neurons	74 nM s^−1^	*
*K*_m,[NE]_ → _DRN_	substrate concentration for the [NE] release in DRN neurons	400 nM	*
*V*_max,[NE]_ → _LHA_	maximum uptake rate for the [NE] release in LHA neurons	74 nM s^−1^	*
*K*_m,[NE]_ → _LHA_	substrate concentration for the [NE] release in LHA neurons	400 nM	*
*V*_max,[5-HT]_ → _DRN_	maximum uptake rate for the [5-HT] release in DRN neurons	1800 nM s^−1^	#
*K*_m,[5-HT]_ → _DRN_	substrate concentration for the [5-HT] release in DRN neurons	170 nM	#
*V*_max,[5-HT]_ → _LHA_	maximum uptake rate for the [5-HT] release in LHA neurons	1800 nM s^−1^	#
*K*_m,[5-HT]_ → _LHA_	substrate concentration for the [5-HT] release in LHA neurons	170 nM	#
*V*_max,[5-HT]_ → _LC_	maximum uptake rate for the [5-HT] release in LC neurons	1800 nM s^−1^	#
*K*_m,[5-HT]_ → _LC_	substrate concentration for the [5-HT] release in LC neurons	170 nM	#
*α*_[Ox]_ → _LC_	rise factor for [Ox] release in LC neurons	0.2314 nM	+
*η*_[Ox]_ → _LC_	decay rate for [Ox] release in LC neurons	0.85 s^−1^	+
*α*_[Ox]_ → _DRN_	rise factor for [Ox] release in DRN neurons	1.405 nM	+
*η*_[Ox]_ → _DRN_	decay rate for [Ox] release in DRN neurons	0.85 s^−1^	+
[5-HT]_*p*,LHA_	per-stimulus [5-HT] release in LHA neurons	12.14 nM	+
[5-HT]_*p*,LC_	per-stimulus [5-HT] release in LC neurons	0.852 fM	+
[NE]_*p*,DRN_	per-stimulus [NE] release in DRN neurons	27.272 nM	+
[NE]_*p*,LHA_	per-stimulus [NE] release in LHA neurons	0.0642 nM	+

In general, the afferent current *I_i_* can consist of several different types of currents mediated by the different modulators and their receptor subtypes. Each of these currents will be determined by the corresponding neuromodulator concentration levels and the receptor affinities (figure 2). For example, suppose a neuromodulator *y* from region *j* induces a current 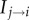 on target region *i*, then we can describe the dynamics of the current by2.3
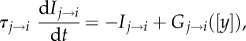
where [*y*] is the neuromodulator concentration level, and 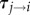 is the effective time constant owing to the applied neuromodulator which can be estimated from experiments. (If there are more than one receptor subtypes mediated by the same neuromodulator over the same brain regions, then we can specify the above variables further, e.g. by defining 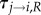 and 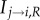 for a receptor subtype *R.*) The value of 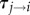 can be deduced from the response dynamics of the induced current (or neural firing rates, if the induced current data are not available) upon infusion of specific neuromodulator at the targeted neurons. The input–output function 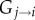 can be described by the sigmoid-like function commonly used in pharmacology [59]2.4
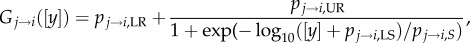
where 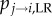 and 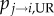 determine the range of the neuromodulatory effect on the currents, and 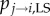 and 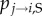 control the lateral shift and the slope of the neuromodulator response current function, respectively. The values of these parameters will be fitted to experimental data through firing rate–neuromodulator concentration or firing rate–current relationships. We used the standard nonlinear regression method, nlinfit from Matlab (The MathWorks Inc., Natick, MA, 2000). This approach particularly allows us to circumvent the complexity of actually simulating the intracellular signal transduction at the post-synaptic neurons. This post-synaptic current depends upon the extracellular neuromodulator release which is, in turn, dependent on the (pre-synaptic) neural population firing rate of the source neurons. Thus, to close the loop in the model, we have to mathematically describe how the release-and-reuptake dynamics are affected by the neural firing rate of the neuromodulator source. We follow a mathematical form similar to that estimated from voltammetric measurements [40]:2.5
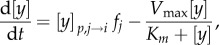
where 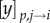 is the per stimulus [*y*] release (at the targeted area *i* from source *j*). The rightmost term in equation (2.5) represents the reuptake rate, and is approximated from the Michaelis–Menten equation. Here, *K*_m_ and *V*_max_ are the Michaelis–Menten constants, with *V*_max_ defined as the maximum uptake rate and *K*_m_ is the substrate concentration where the uptake proceeds at half of the maximum rate. The value for both of these parameters can be obtained from experiments [40]. In voltammetry experiments, *f_j_* is typically an artificially applied high current stimulus frequency to stimulate the release of *y*. However, following our previous work [60], we can redefine it as the neural firing frequency of the neuromodulator source. Hence, the value of 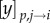 has to be adjusted from that in voltammetry experiments, and the exact value in the model can be obtained by constraining the overall basal activity levels of the system to be within the observed experimental ranges (see below).

For the case of Ox, there is a lack of available experimental data, particularly its release and reuptake dynamics. Hence, we adopt the simplest mathematical form to describe the [Ox] dynamics [60], with only two parameters:2.6
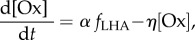
where *α* is the rise factor and *η* is a constant decay rate, and both considered to be free parameters. The value of *α* is selected, so that the release of [Ox-A/B] at DRN or [Ox-A] at LC is close to the observed basal value (table 1).

A summary of the general model construction process is summarized in figure 2. Such a modelling approach can allow multiple brain regions to be constructed, simulated and analysed. (See Methods for a simpler approach when only two brain regions are considered.) Overall, we have proposed an efficient and scalable approach by incorporating neuromodulator properties and dynamics into traditional firing-rate-type models. We shall next apply this approach to develop a neural circuit model involving multiple interacting neuromodulators.

### 2.2. An example with three interacting neuromodulators

We shall now demonstrate, as an example, the steps towards developing a neural circuit model of three interacting neuromodulator systems (lateral hypothalamus, DRN and LC) through three corresponding neuromodulators (Ox, 5-HT and NE), based on available experimental data and equations (2.1)–(2.6). These brain regions were chosen mainly because (i) they consist of different neuromodulator systems that can directly influence each other, (ii) they were targets of existing drugs, and (iii) we can demonstrate how one could model with incomplete information. It is important to bear in mind that different datasets from various experiments are used for model construction and validation.

First, the frequency–current (*f* − *I*) curves for neurons from the three brain regions are determined according to the available electrophysiological data (*f* − *I* curves and typical baseline firing rate ranges). Using the threshold-linear function (equation (2.2)), the fitted parameter values for the LC's NE neuronal *f* − *I* curve are 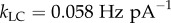
*,* 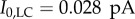 and 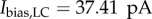 [46]; that for DRN's 5-HT neuron are 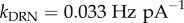, 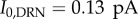 and 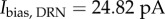 [10,44,45]; and that for LHA's Ox neuron are 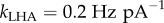, 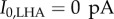 and 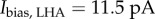 [43].

**Figure 3.**
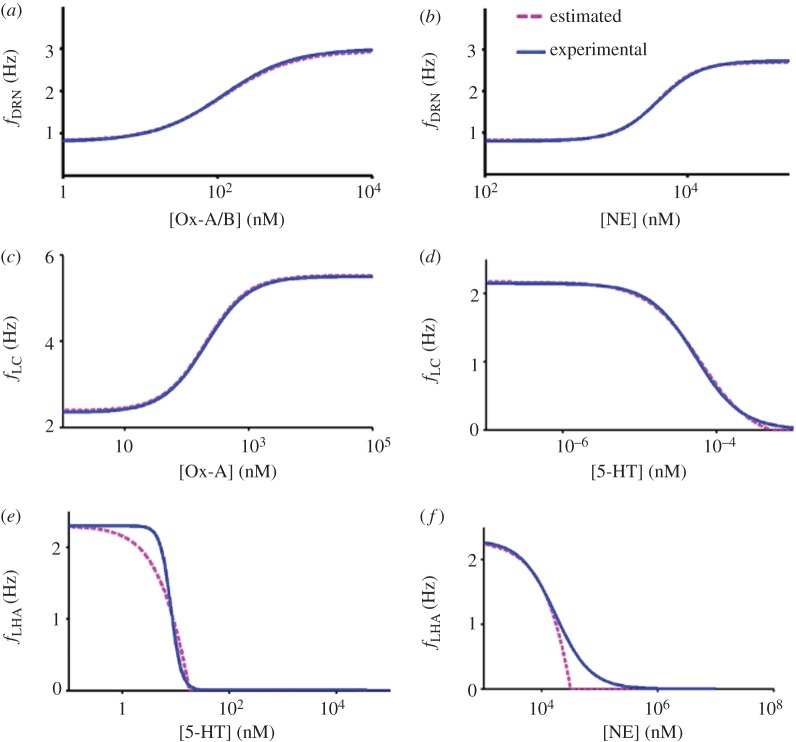
Fitted input–output functions. (*a,b*) Effects of concentrations [Ox-A/B] and [NE] on DRN neurons. (*c,d*) [Ox-A] and [5-HT] on LC neurons. (*e,f*) [5-HT] and [NE] on LHA neurons. Estimated function (pink) is based on *f–I* curves and current input–output functions.

Next, parameters of the induced currents and associated *G* functions are fitted to the experimental data through the concentration–response relationships for the change in firing rate induced by the neuromodulator. For example, the total afferent current induced by neuromodulators on DRN's neurons can be rewritten as a sum: 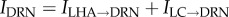. The terms on the right are the currents owing to Ox-A/B from LHA and NE from LC (for simplicity, we ignore the current owing to autoreceptors and interneurons within each brain region, see [17,61]). The dynamics for each induced current are dependent on the concentration of the neuromodulator. The Ox-induced current on DRN neurons (when NE-induced current on DRN is clamped) can be described by2.7
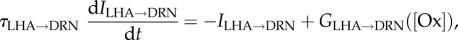
where 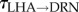 is the effective time constant owing to the injected Ox deduced from experiments. The 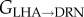 function (from equation (2.4), with units of currents) parameters are fitted to experimental data [53], such that 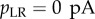, 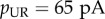, 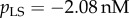, 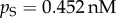 and 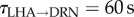. With these values, we obtain the best fit for the overall firing rate–concentration function (figure 3*a*), having the induced currents becoming an implicit function.

Similarly, we can obtain the parameter values for the other currents: 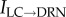, 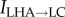 and 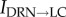. The fitted parameter values for 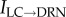 are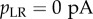, 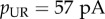, 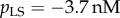, 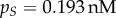 and 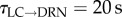 (figure 3*b*) [55]. Parameter values for the incoming current from LHA to LC neurons 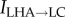 are found to be 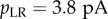, 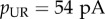, 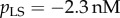, 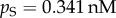 and 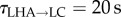 [62] (figure 3*c*). For *I*_DRN→LC_: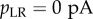, 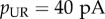, 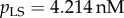, 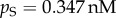 and 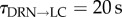 (figure 3*d*). With these parameter values, we were able to obtain reasonable fits with respect to experiments for the various input–output functions.

We now proceed to model the effects of 5-HT on LC's NE neurons. However, there is a lack of experimental data on the direct effects of 5-HT on LC's NE neurons (experiments typically focused on how different 5-HT_2_ receptor agonists affect the firing rate of LC neurons [63]). Thus, to estimate the 5-HT-dependent input–output function of firing frequency, we approximate the input–output function from other experimental data by restricting the basal activities to [5-HT] ∼ 0.11 *f*_M_ and *f*_LC_ ∼ 2.15 Hz (table 1). We considered the same sigmoidal shape (as defined in equation (2.4)) for all the neuromodulator-dependent firing-rate function which is defined as2.8
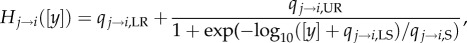
where 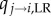 and 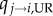 determine the range of the neuromodulatory effect on the firing rate, and 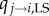 and 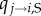 control the lateral shift and the slope of the neuromodulator concentration-dependent firing-rate function 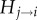, respectively. Considering these baseline values, the estimated parameter values are 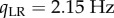, 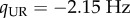, 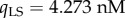 and 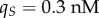 [42].

Similar to the 5-HT modulating effect on LC, the 5-HT response direct firing–frequency curve was not available for LHA neurons. Thus, we approximate the input–output function from other experimental data [37,47] and restricting the basal activities to [5-HT] ∼ 1.6 nM and 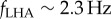. Then, the estimated parameter values are 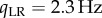, 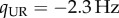, 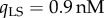, 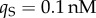 [36]. Then, we approximated the parameters for 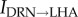 to be: 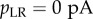,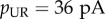, 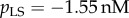 and 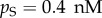. With these values, we observe that there is no Ox neuronal firing even for 10 µM of [5-HT] (figure 3*e*). This is due to the strong inhibition caused by the induced inward GIRK current (approx. 32 pA), which eventually saturates (at approx. 35 pA) for higher [5-HT] levels*.*

As the model has a hard threshold in the *f–I* curve, there is a sharp change within the 10–100 nM of [5-HT]. Thus, we could not obtain a perfect fit for the functions 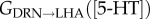 and hence the 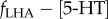 curve. Similarly, the fitted parameters for the NE-induced GIRK currents on LHA, 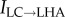, are 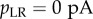, 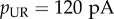, 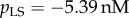, 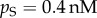 and 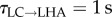 [56]. We encounter a similar issue for higher [NE] level, i.e. no perfect fit for 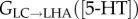 (figure 3*f*).

Compared with the effect of 5-HT on the target areas, estimating the parameter values (*q*'s) for the effect of NE/Ox on the target areas was relatively straightforward. As experimental data related to neuromodulator-dependent firing-rate input–output function were available, we approximated the *q*-values for the Ox effect of DRN as 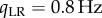, 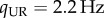, 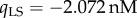, 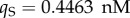 [62]. *q*-values for the Ox effect of LC are 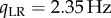, 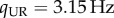, 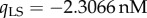, 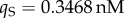 [62]. Similarly, *q*-values for the NE effect of LHA are estimated as 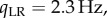 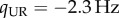, 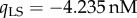, 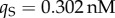 [56]. For NE effect on DRN they are 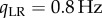, 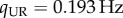, 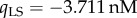, 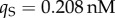 [64].

**Figure 4.**
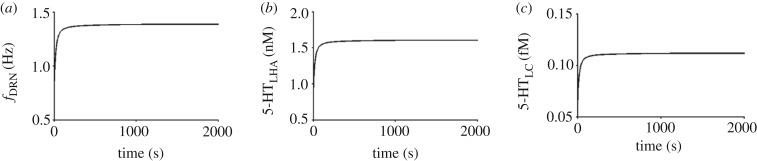
Single trial activity dynamics under baseline condition. (*a*) Firing rate of DRN neural population. (*b,c*) Concentration level of 5-HT in the LHA and LC areas.

After determining the input–output functions and dynamics for all the currents, the final step is to integrate all three brain regions and their interactions. In general, the activities for the combined system will be different from the individual isolated systems. Thus, the baseline activities of the coupled system will be different from that observed from the individual systems. However, the remaining set of parameters, the neuromodulator release per stimulus frequencies, i.e. the [*y*]*_p_*'s, can be adjusted to resolve this. We found that for values of 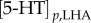, 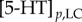, 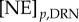 and 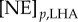 at 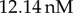, 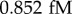, 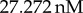 and 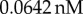, respectively, and Ox rise factor and decay rate (*α* and *η*) for DRN are 1.405 and 0.85 l s^−1^ while for LC are 0.2314 nM and 0.851 s^−1^, the overall basal firing rates and concentration levels are reasonably within the experimental ranges (table 1). Note that the baseline activities are obtained after sufficiently long simulation to attain their (stable) steady states (see figure 4 for a sample trial).

After successfully constructing the LHA–DRN–LC circuit model, we shall demonstrate simulating neuropharmacological drug effects in the system. In particular, we shall focus on effects of Ox-1 receptor antagonist (SB-334867-A), SSRIs and/or SNRIs on the LHA–DRN–LC circuit.

### 2.3. Drug effect simulations

Pharmacologically, antagonists can be classified into two categories: competitive and irreversible antagonists [65]. Pre-treatment or application of competitive antagonist can shift the baseline dose–response curve horizontally. This shift towards the higher doses (of neurotransmitter) increases the effective dose (ED_50_) value of the dose–response curve (where 50% of the maximal response of the dose is being observed). Conversely, application of an irreversible antagonist can cause shifts in the maximum range of the antagonist effect and does not affect the ED_50_ value [66].

Ox-1 receptor antagonists have been suggested to encourage sleep, as well as treatment and prevention of many psychiatric disorders [67]. In particular, the Ox-1 receptor antagonist, SB-334867-A, acts as a competitive antagonist which rightward shifts the Ox-A response curve in 5-HT and NE neurons in DRN and LC [62,68]. Thus, we can easily incorporate the effect of SB-334867-A, by simply laterally shifting the function 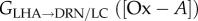.

Selective serotonin/norepinephrine reuptake inhibitors (SSRIs/NRIs) are some of the commonly known pharmacological agents that are used for the treatment of various psychiatric disorders. The basic (acute) actions of these drugs are similar: primarily to increase the extracellular concentration level of their respective neuromodulator concentration by inhibiting the uptake process and reduce the synaptic clearance in the extracellular space [69]. There have been numerous studies conducted to understand the effects of uptake inhibitors on [5-HT] uptake [69,70]. In particular, John *et al.* [70] show that monoamine uptake inhibitors can affect the values of the Michaelis–Menten constants *K*_m_ and *V*_max_ in the limbic part of the brain. For example, 10 µM of fluoxetine, an SSRI (when applied to the substantia nigra area), increased the value of *K*_m_ by about a factor of 5 but did not alter the value of *V*_max_ significantly [69]. Thus, to incorporate the influence of SSRIs/NRIs in our model, we can mimic the different doses of SSRIs by varying the different values of *K*_m_. For simplicity, our model will ignore chronic or other long-term secondary actions such as receptor density changes.

**Figure 5.**
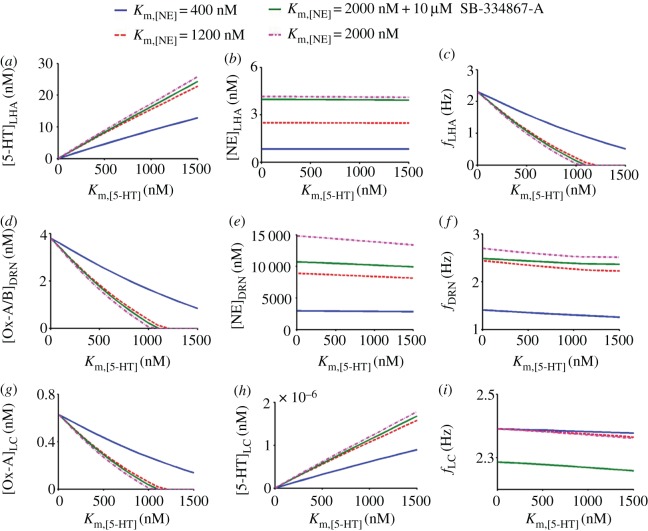
Effects of substrate concentration factor *K*_m,[5-HT]_ and *K*_m,[NE]_, and 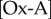 antagonist SB-334867-A on the firing rates and concentration levels in the circuit. Each panel varies both *K*_m,[5-HT]_ and *K*_m,[NE]_ values to simulate the effects of drugs and their combinations. *K*_m,[NE]_ = 400 nM (control basal value).

As we increase the value of 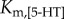 (mimic SSRI), [5-HT] linearly increases in both the targeted areas LHA and LC (figure 5*a,h* solid blue). This increase in [5-HT] level causes a significant decrease in *f*_LHA_ (figure 5*c*, solid blue), which is consistent with experimental findings [36]. This in turn causes a decrease in [Ox-A/B] levels in the DRN and LC areas (figure 5*d,g* solid blue). Interestingly, because of the network effect, there is a subsequent decrease in *f*_DRN_ (figure 5*f*, solid blue), consistent with [71] even when we did not incorporate any inhibitory 5-HT autoreceptors [72,73]. However, 5-HT's effect on LC's NE neurons is minimal, consistent with [74], and therefore, *f*_LC_ does not alter the [NE] levels in the DRN and LHA significantly (figure 5*i*, solid blue). These effects remained to be validated in the intact brain.

Next, we simulate the combined effects of SSRIs and NRIs, by increasing the value of *K*_m,[NE]_ to three and five times its control value (400 nM) while varying *K*_m,[5-HT]_ as previously. The model shows that for higher values of *K*_m,[NE]_ more [5-HT] and [NE] are released in the targeted areas in the LHA and LC when compared with controls (figure 5*a,h* dashed red and dotted-dashed pink). This suggests that other than 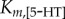, [NE] release in DRN also helps stimulate the release of [5-HT] in these targeted areas, consistent with [75]. This rise in the [5-HT] level significantly affects *f*_LHA_, 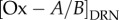, 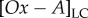, 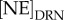 and 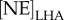 while there is little impact on *f*_LC_ (figure 5*b–e,g,i*).

Finally, to assess the combined effect of SSRIs, NRIs and SB-334867-A, we set 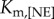 to be five times the control value and mimic the influence of 10 µM SB-334867-A on DRN and LC (by changing the *p*_LR_ value from 3.8 to 2 pA, *p*_UR_ from 54 to 51 pA, shift factor *p*_LS_ from −2.3 to −4.192 nM, and slope factor *p*_S_ from 0.341 to 0.592 in LC). For DRN, *p*_LS_ is changed from −2.08 to −2.97 nM and *p*_S_ from 0.452 to 0.367. We find that this triple-drug combination can cause a further decrease in the *f*_DRN_ and *f*_LC_, and a substantial reduction in [NE]_DRN_ levels (figure 5*e,f,i* solid green), whereas the rest are not significantly affected by the addition of SB-334867-A (figure 5*a–d,g,h* solid green).

### 2.4. Software for model simulation and visualization

**Figure 6.**
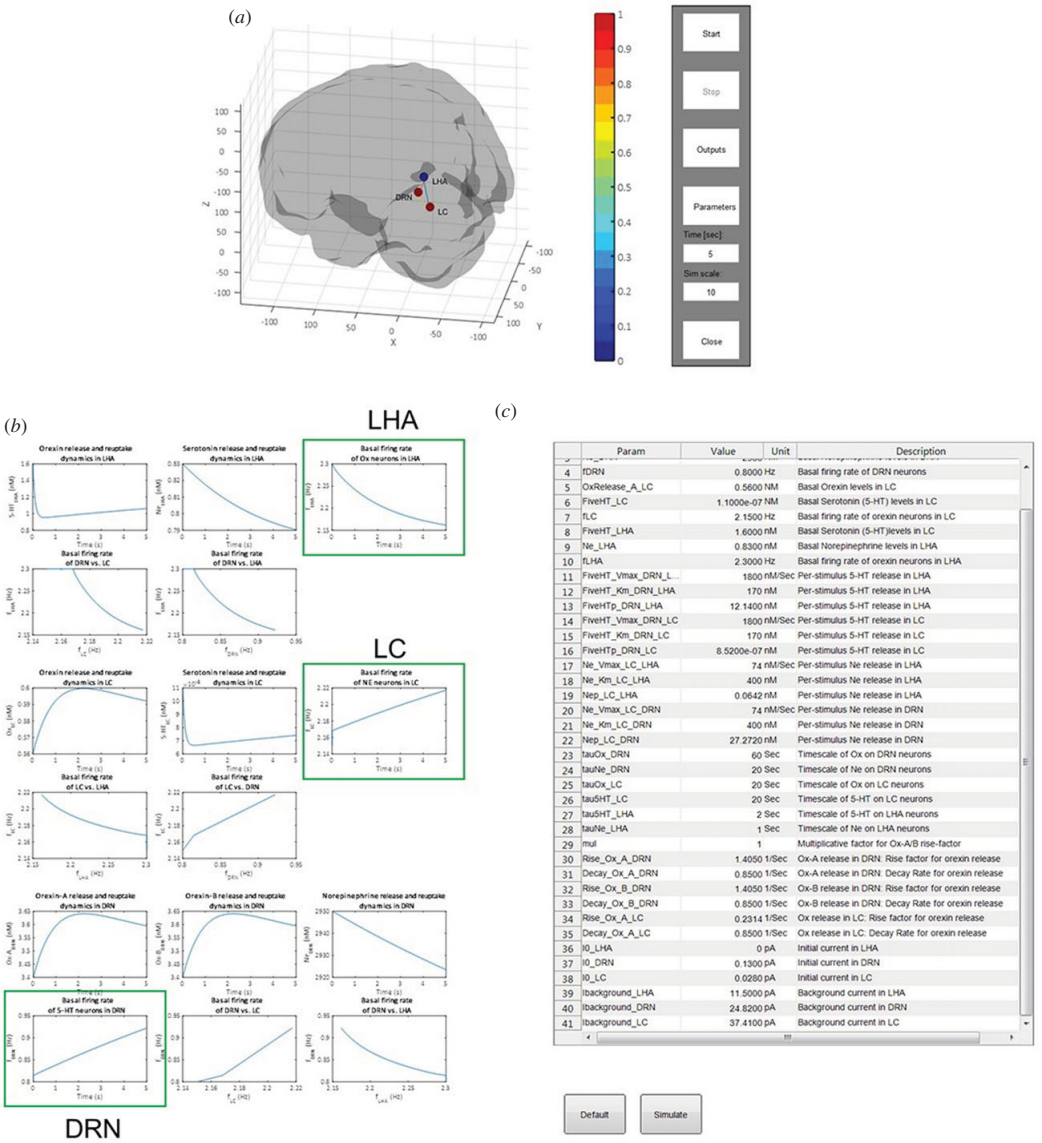
Screenshot of the NModC software. A user-friendly GUI of neuromodulator neural circuit model that can simulate, analyse, visualize and edit. (*a*) Users can run the model to visualize the results within a rotatable three-dimensional glass brain after pressing the ‘Start’ button. The user can stop the simulation using the ‘Stop’ button. Simulation time parameters can be controlled using ‘Time’ and ‘Sim scale’, and the GUI can be closed using ‘Close’ buttons. (*b*) Model variables' time courses and their mutual relationships can be observed using the ‘Outputs’ button. (*c*) Model variables' exact values can be found and model parameters edited upon pressing the ‘Parameters’ button. ‘Default’ returns to default model parameters and ‘Simulate’ re-runs the model after editing the parameter values.

Using Matlab, we have designed and developed a software, called ‘NModC’ (neuromodulator circuit), with friendly graphical user interface (GUI) for simulation, analysis and visualization of the types of models described. The software is easy to use, and can easily be generalized to additional brain regions, other neural subpopulations and neuromodulator types. The user can visualize the activities of multiple brain regions dynamically and simultaneously. These brain regions are embedded in a rotatable three-dimensional glass brain using standard Montreal Neurological Institute (MNI) coordinates (figure 6*a*). The user can also further specify brain regions of interest to find the dynamical variables' time courses and mutual relationships (figure 6*b*) for more detailed analysis. The model parameters can be easily altered to visualize the variation in the steady-state values of the transients of neuromodulator concentration level and firing rates, and can also compare the firing rates of the two brain regions (figure 6*c*). Further details are described in the Methods section, and the software is available at https://github.com/vyoussofzadeh/NModC.

## 3. Discussion

In this work, we have proposed a new computational modelling framework for incorporating essential biological features of neuromodulation in neural circuits. This provides a computational platform to link from low-level neurobiology to large-scale brain activities.

Our framework is based on the population-averaged firing-rate type of model which has model parameters constrained by neurobiology. This is to be compared with other firing-rate-type models without such constraints [14–16]. The model integrates pharmacological and electrophysiological data from separate experimental studies to constrain the input–output neuronal functions, and also the timescale and profile of the effective neuromodulator-induced currents. Another key difference in our modelling approach is the consideration of the release-and-reuptake/decay dynamics of the extracellular neuromodulator concentration level that can be inferred from voltammetry. By doing this, we circumvented modelling the complex intracellular biochemical processes, but instead, directly modelled the concentration-dependent effect on neural firing rate activity based on pharmacological–electrophysiological data.

In particular, to allow our approach to be generalizable to multiple interacting brain regions, we introduced neuromodulator-induced currents to bridge the gaps between neuromodulator concentration levels and targeted neural firing rate activities—afferent influences from different brain regions can be accounted for by their summed currents. The timescale and response curve of the neuromodulator concentration-dependent currents were constrained by data from combined pharmacological and electrophysiological experiments.

We demonstrated our modelling approach with the example of a neural circuit that involves three mutually interacting brain regions (LHA, DRN and LC), which are also sources of three different neuromodulators: orexin, serotonin and NE, respectively. The transient and steady-state dynamics of the experimentally measurable variables (neural firing rates and neuromodulators' extracellular concentration levels) could be easily simulated. In particular, our model supported the coexistence of the observed (steady-state) baseline firing rates and neuromodulator levels found in separate experiments.

An important application of our model was the prediction of the effects of neuropharmacological drugs on neural circuits. We simulated the effects of SSRIs, NRIs and Ox-1 receptor antagonist on the LHA–DRN–LC model. We first showed that SSRIs could have a wide effect on the neural circuit, except the LC–NE system. Interestingly, SSRI could inhibit the DRN (decrease in *f*_DRN_), the source of 5-HT, even though we did not implement its inhibitory autoreceptors. This effect was essentially owing to the direct effects on serotonin heteroreceptors on the LHA and LC, which in turn inhibited the DRN. Similar circuit-based effects could be explained for the addition of NRIs and/or Ox-1 receptor antagonists.

The constructed LHA–DRN–LC circuit model turned out to be dominated by a unidirectional influence between any pair of interacting brain areas (figure 5). Hence, these result in monotonic relationships (either increased or decreased) as the model parameters (e.g. *K*_m_'s) were varied. However, this need not generally be the case. For example, a more balanced (especially excitatory–inhibitory) coupled network could easily lead to emergent circuit oscillations or even non-monotonic effects [8,76]. In the latter case, it might then be possible to search for the optimal drug dosage. In fact, we had shown evidence of such nonlinear emergent behaviour when the model incorporated autoreceptors and non-principal (e.g. inhibitory GABAergic) interneurons (to mediate indirect connections) [17]. Moreover, the excitatory–inhibitory balance of the network can also be influenced by the co-release of the neurotransmitters (e.g. glutamate) [77,78]. In this case, our framework can still accommodate this by introducing additional dynamical equations to describe the effects of glutamate or GABA (for the same pre-synaptic firing rate).

Our work has also shown that administration of multiple drugs (serotonin/NE reuptake inhibitors and Ox-A antagonist) simultaneously can be simulated in neural circuits to search for the optimal mixture of drugs. However, the results remain to be validated as there is a lack of such work done in experiments. Hence, this will form model predictions that can help experimentalists in designing future studies. For example, multielectrode array *in vivo* recordings can be designed to study the wide-ranging effects of drugs on different brain areas. It would also be interesting to use the model to minimize the side effects of drugs, which is an important issue in neuropharmacology.

Our modelling framework is scalable to incorporate multiple brain regions and hence can be used to study large-scale brain effects. This includes studying the changes in REM/non-REM stages or sleep–wake cycle [15,16], cortical dynamics [8] and cognitive–emotional processing [8,76]. This would require extending our current GUI software by including cortical brain structure and their connectivity with the neuromodulator sources. Thus, these models could potentially reveal insights into the relationships among various brain and behavioural disorders (depression, addiction, antidepressants and sleep disorders). Importantly, neuroimaging data, especially from positron emission tomography and functional magnetic resonance imaging, could potentially be incorporated into our modelling framework, bridging across multiple scales and modalities, similar in spirit to the popular dynamic causal modelling approach [79]. Interestingly, recent whole brain molecular imaging (functional magnetic resonance imaging (MRI)) of serotonin transporter to characterize 5-HT dynamics in humans before and after (e.g. SSRI) drug administration is now possible [80], opening up another possible application of our modelling framework.

In summary, we have proposed a promising new computational modelling framework that can integrate various experimental neurobiological data into a computationally efficient large-scale neural circuit model for simulating, testing and predicting the effects of multiple endogenous neuromodulators and neuropharmacological drugs.

## 4. Methods

### 4.1. A simpler modelling approach for modelling two brain regions

Note that if one is only interested in the mutual interactions of two brain regions, then one may ignore the induced current implementation step (figure 2, second column), and directly model the influence of [*y*] on the firing rates *f_i_* [60], i.e.4.1
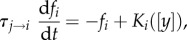
where 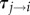 is the effective time constant owing to the injected neuromodulator *y* on the *i*th neural population. *K_i_*([*y*]) can follow a similar form as the *G* function in equation (2.4).

### 4.2. Model parameter and baseline values

A summary of the LHA–DRN–LC model parameter values and baseline activities are shown in table 1.

### 4.3. Numerical simulations

The neural circuit model simulation for the interaction of the three brain areas is computed by using the forward Euler numerical integration method which is applied to the set of the first-order differential equations. A time step of 1 ms was used. Smaller time steps were tested without affecting the results. These simulations can also be extended to other (e.g. second-order or fourth-order Runge–Kutta) numerical schemes. Simulations were run until stable steady states are obtained.

### 4.4. Graphical user interface software

To begin the software, the user presses the ‘Start’ button on the starting window of the GUI. This will result in the model outputs of the interacting brain regions (figure 6*a*). The outputs appear in the form of normalized neural (firing rate) at the specified brain regions, e.g. red colour represents relatively higher activity, whereas blue colour represents lower activity. The range of the colour map is based on the absolute range of [0 255] Hz. These colours of activity can change over time, reflecting their dynamics. The regions are embedded in locations based on the MNI coordinates in a glass brain. Three-dimensional rotation of the brain is also allowed in the software. Although the structure of the glass brain is currently based on normal human MRI data, it can be easily replaced by an animal (e.g. rodent) glass brain using the appropriate brain atlas.

Once the model is converged after simulation, upon clicking on the ‘Outputs’ button in the starting window, the dynamical variables for the selected regions will appear in a new window (figure 6*b*). The variables are the (absolute) neural firing rates and neuromodulator concentrations of the selected brain regions. Both the individual variable's temporal dynamics and mutual relationships between the variables can be plotted. Upon clicking the ‘Parameters’ button in the starting window, a new window appears in which the model parameter values and the initial numerical values of the variables are shown. The model parameter values can be edited within this window. Once this is done, the user can resimulate the new model by pressing the ‘Simulate’ button within the same window. This generates the transients of baseline firing rates and concentration levels and shows the relationship among them (e.g. firing rates). For default values, all the transient activities eventually attain their stable steady states. These steady-state values of the system parameters vary as we change the model parameters. For example, to mimic the complex drug effects of SSRIs, *K*_m_ is varied and corresponding changes in the steady-state values are analysed separately (see §2.3). If a mistake is made, then the user can always retrieve back the initial values of the parameters by clicking on the ‘Default’ button.
